# Supplementary material for: Quantification of the Helical Morphology of Chiral Gold Nanorods
Source: ACS Mater Lett. 2022 Mar 8;4(4):642–9. doi: 10.1021/acsmaterialslett.2c00055 (PMC8986031; doi:10.1021/acsmaterialslett.2c00055)
Supplement: Supplementary file 1 — tz2c00055_si_001.pdf [file tz2c00055_si_001.pdf]

## Supporting information

# Quantification of the helical morphology of chiral Au nanorods


Wouter Heyvaert[1], Adrián Pedrazo-Tardajos[1], Ajinkya Kadu[1], Nathalie Claes[1], Guillermo González-Rubio[2,3], Luis M. Liz-Marzán[2,4,5], Wiebke Albrecht[1,6]*, Sara Bals[1]*

[1] EMAT and NANOlab Center of Excellence, University of Antwerp, 2020 Antwerp, Belgium

[2] CIC biomaGUNE, Basque Research and Technology Alliance (BRTA), 20014 Donostia-San Sebastián, Spain

[3] Physical Chemistry Department, University of Konstanz, Universitätsstraße 10, Box 714, 78457 Konstanz, Germany.

[4] Centro de Investigación Biomédica en Red de Bioingeniería Biomateriales, y Nanomedicina (CIBER-BBN), 20014 Donostia-San Sebastián, Spain

[5] Ikerbasque, Basque Foundation for Science, 48009 Bilbao, Spain

[6] Center for Nanophotonics, AMOLF, Science Park 104, 1098 XG Amsterdam, The Netherlands


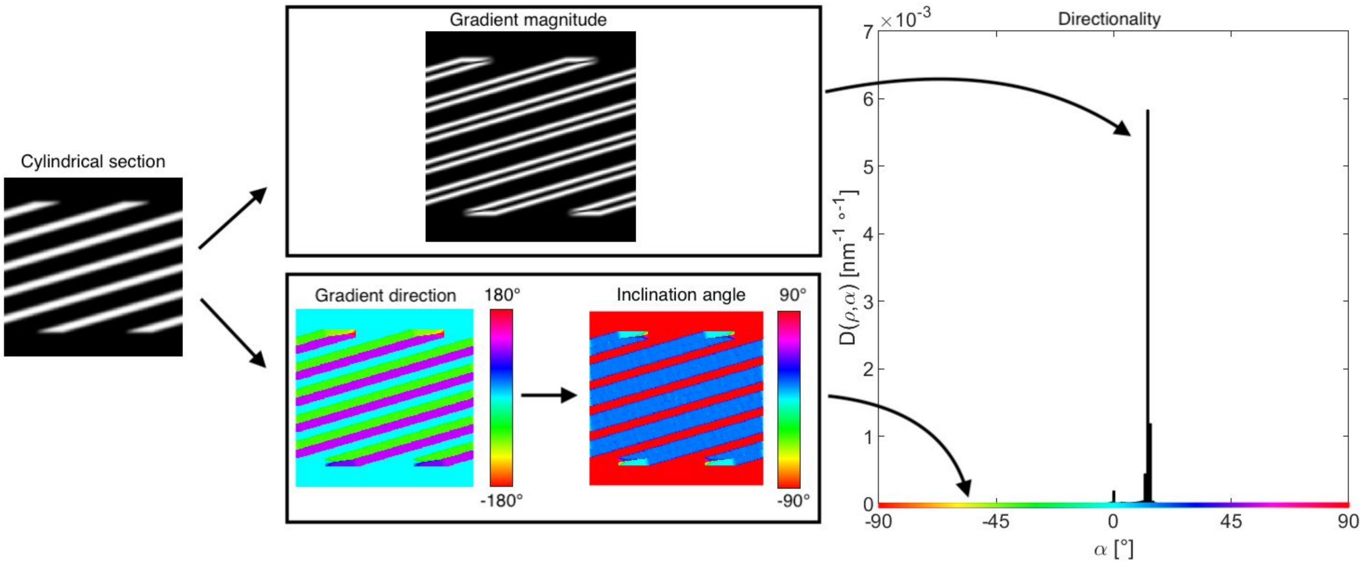


**Figure S1.** Visualization of the calculation of the directionality histogram for a single cylindrical section. First the gradient is calculated from a cylindrical section, which yields two images: a gradient magnitude and a gradient direction. The inclination angle for each pixel can be calculated based on the gradient direction. Finally, the inclination is used to select the correct bin in the directionality histogram for each pixel and the value of the gradient magnitude in that pixel is added to that bin. This process is repeated for each pixel of each cylindrical section.

## 1. Normalization of directionality

To calculate the directionality for a 2D image, the gradient is first normalized such that $\sum_i \|\vec{g}_i\| = 1$, with the sum going over all pixels in the image and $\vec{g}_i$ is the gradient vector of the $i$-th pixel. Consequently, the directionality will also be normalized such that the sum of all bins equals one. However, to combine the directionality of all cylindrical sections, the metric of cylindrical coordinates ($dx\, dy\, dz = \rho\, d\rho\, d\theta\, dz$) must be considered. Furthermore, even though the directionality can be calculated for each cylindrical section separately, the intensity of different features should still be comparable between sections. This means that one cannot simply normalize the gradient of each cylindrical section separately, but the gradients of all sections must be normalized together. If $f_\rho(\theta,z)$ is a cylindrical section through a particle at radius $\rho$ and $\vec{g}_i^{(\rho)}$ is the amplitude of its gradient at pixel $i$, then the normalization must be $\sum_\rho \sum_i \rho \cdot \|\vec{g}_i^{(\rho)}\| = 1$.

## 2. Algorithm and computational efficiency

To calculate the helicity function, the 3D volume must first be converted into cylindrical coordinates, which can be achieved via linear interpolation. For a volume of $N = n \times n \times n$ voxels, the computational complexity of this algorithm is $\mathcal{O}(N)$. Next, the helicity function can be calculated using the following $\mathcal{O}(N)$ procedure:

```
For each voxel in a cylindrical volume
        Calculate the gradient in the voxel in its cylindrical section
        Calculate the inclination angle and gradient magnitude for the voxel
        Add the gradient magnitude of the voxel to the bin corresponding to the inclination angle and radius in the
        2D directionality histogram
End
Normalize the directionality such that the sum of all bins is 1
Calculate the helicity function by subtracting the bins in the directionality at negative inclination angles from the bins
at the corresponding positive inclination angles
```

The gradient is calculated using Sobel filters in the cylindrical sections, which yield the gradient in the $\theta$- and z-directions as a vector. The gradient magnitude is then calculated as the norm of this gradient vector and the inclination angle is calculated using the angle between the gradient vector and the x/y-plane.

We also present two "extensions" of this method. A single-value total helicity $H_{total}$ can be used to identify the total helicity of a nanoparticle and to compare the helicity of different nanoparticles. This value is calculated as the sum over the full helicity function. The number of bins for the inclination angle $\alpha$ is fixed, and the number of bins for the radius $\rho$ equals the number of cylindrical sections in the cylindrical volume. This means that the sum over the helicity function $H(\rho,\alpha)$ scales with $\mathcal{O}(N^{1/3})$ such that the calculation of the total helicity $H_{total}$ scales with $\mathcal{O}(N)$. A helicity map can also be created by calculating $H_{total}$ in a small window around each voxel in cylindrical coordinates. Since the window size is fixed, this method also scales with $\mathcal{O}(N)$.

The computation time on a desktop PC (AMD Ryzen 7 1700 CPU and 16GB of RAM) for the different steps of our method and for different sizes of the input volume is presented in Figure S2. For the Au NRs analyzed in the main text, a volume of about $300 \times 300 \times 300 = 2.7 \cdot 10^7$ voxels was sufficient to capture all details. Full results could therefore be retrieved in under five minutes. However, for even larger volumes such as a volume of $500 \times 500 \times 500 = 12.5 \cdot 10^7$voxels, full results could be retrieved in less than 30 minutes. When only calculating the helicity function $H(\rho,\alpha)$ and total helicity $H_{total}$, the results for the largest volumes were obtained in less than 1 minute. It should also be noted that, even if finer detail is required, an implementation to calculate the helicity map could be created to improve the computation time by a GPU.

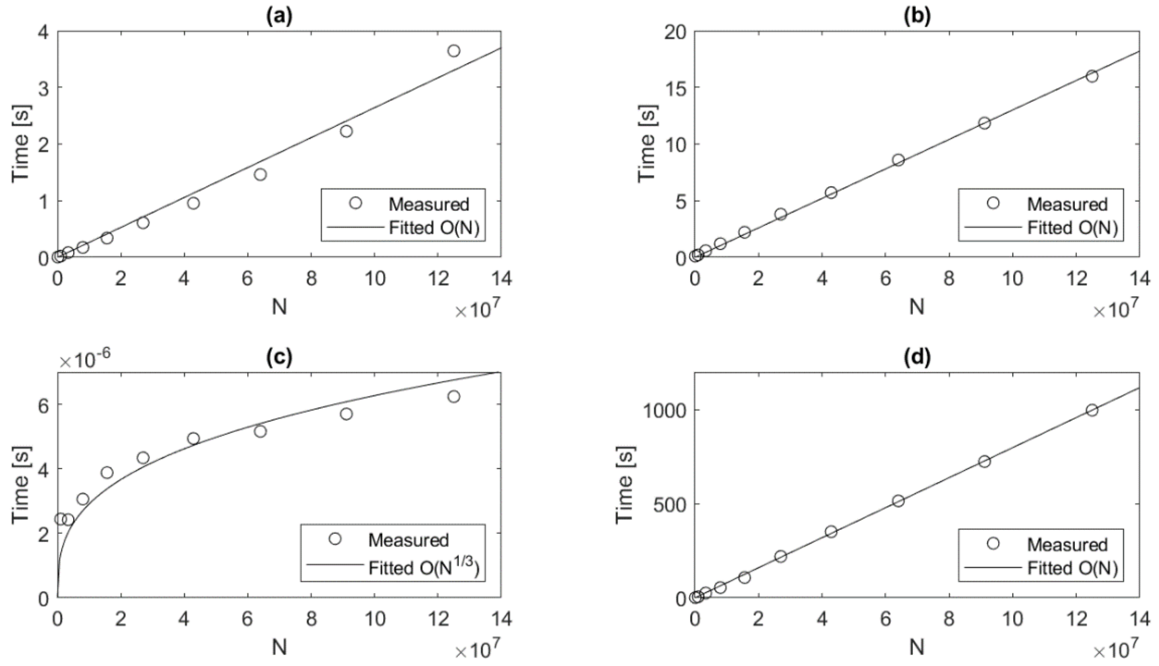


**Figure S2.** Calculation time and fitted curves of the expected complexity for: (a) interpolating a volume of $N = n \times n \times n$ voxels on a cylindrical grid; (b) calculating the helicity function $H(\rho,\alpha)$ for that volume; (c) calculating the total helicity $H_{total}$ after the helicity function has already been calculated; and (d) creating a helicity map for that cylindrical volume. Calculations were carried out using a vectorized implementation in Matlab on a desktop computer with an AMD Ryzen 7 1700 CPU and 16GB of RAM.

### 3. Synthesis details

**Materials.** All starting materials were obtained from commercial suppliers and used without further purification: 1-decanol (n-decanol, 98%), cetyltrimethylammonium bromide (CTAB, ≥99%), (R)-(+)-1,1′-Bi(2-naphthol) ((R)-BINOL, 99%), (R)-(+)-1,1′-binaphthyl-2,2′-diamine ((R)-BINAMINE, 99%), (S)-(-)-1,1′-binaphthyl-2,2′-diamine ((S)-BINAMINE, 99%), hydrogen tetrachloroaurate trihydrate ($HAuCl_4 \cdot 3H_2O$, ≥99.9%), silver nitrate ($AgNO_3$, ≥99.0%), L-ascorbic acid (≥99%), L-cysteine (97%, )hydrochloric acid (37%) and sodium borohydride ($NaBH_4$, 99%) were purchased from Aldrich. Cetyltrimethylammonium chloride (CTAC, 99%) was purchased from ACROS Organics. Nanopure water (resistivity >18.2 MΩ·cm at 25 °C) was used in all experiments.

**Synthesis of nanorod seeds.** Gold nanorods were prepared using the multistep seed-mediated growth method with minor modifications, as previously described:[1]

- *Synthesis of 1–2 nm gold seeds:* 20 mL of a freshly prepared n-decanol/CTAB (13.5 mM/ 50 mM) solution was placed in a 50 mL glass beaker at 25–27 °C and then 200 µL of $HAuCl_4$ (0.05 M) and 100 µL of ascorbic acid (0.1 M) were added. After 1–2 min, 800 µL of a freshly prepared 0.02 M $NaBH_4$ solution was injected under vigorous stirring into the colorless solution (1000 rpm using a PTFE plain magnetic stirring bar: 30 × 6 mm, at 25–27 °C). A brownish-yellow solution was obtained, which was aged for at least 60 min at 25–27 °C (using a water bath) prior to use.
- *Synthesis of small anisotropic seeds (21 nm long, 7.5 nm wide):* In a 500 mL Erlenmeyer flask, 3.0 mL of $HAuCl_4$ (0.05 M), 2.4 mL of $AgNO_3$ (0.01 M), 21.0 mL of HCl (1 M), and 3.9 mL of ascorbic acid (0.1 M) were added to 300 mL of a freshly prepared n-decanol/CTAB (13.5 mM/ 50 mM) solution at exactly 25 °C (using a water bath). Under stirring, 18 mL of the seed solution was then added, and the mixture was left undisturbed for 4 h at 25 °C. The obtained small gold nanorods were centrifuged at 14000–15000 rpm for 45–60 min using 2 mL Eppendorf tubes. The supernatant was discarded, and the precipitate was collected and redispersed in 30 mL of a CTAB (10 mM) solution. The concentrated gold nanorod solution was centrifuged again at 14000–15000 rpm for 45–60 min and the precipitate was redispersed in 30 mL of a CTAB (10 mM) solution. This step was repeated one more time. Gold concentration was fixed to 4.65 mM (abs at 400nm: 1.0, optical path: 0.1 cm) by adjusting the volume of the CTAB solution added to redisperse the small nanorods precipitated in the last centrifugation step.
- *Gold nanorods of 29 nm in width and 130 nm in length:* In a 250 mL Erlenmeyer flask, 100 mL of n-decanol/CTAB (13.5 mM/ 50 mM), 1.0 mL of $HAuCl_4$ (0.05 M) and 1.5 mL of $AgNO_3$ (0.01 M) were mixed under magnetic stirring. The mixture was kept undisturbed for an hour at 16 °C (using a Julabo F-25 refrigerator/heating circulator). Then, 3 mL of HCl (1M), 800 µL of ascorbic acid (100 mM) and (once the mixture turned colorless) 45 µL of small gold nanorod solution were added under stirring. The mixture was kept undisturbed overnight at 16 °C for growth of gold nanorods. Finally, the obtained nanorods were centrifuged at 3000-6000 rpm (10-15 min) and redispersed in 10 mL of CTAC (10 mM). This step was repeated twice to remove excess CTAB.

**Growth of chiral gold nanoparticles.** Gold nanorods were prepared as previously reported:[2]

- *Preparation of chiral co-surfactant/CTAC solution:* In a 50 mL glass vial, 35.55 mg of BINAMINE (35.79 mg in the case of BINOL) and 1600 mg of CTAC were weighed. Then, 50 mL of water was added, and the mixture was vigorously stirred at 60 ºC until complete dissolution of the co-surfactant (ca. 3h). The solution was then allowed to cool down to room temperature before further use. The concentration of CTAC and co-surfactant were 100 mM and 2.5 mM, respectively.
- *Washing of the nanoparticles:* To properly remove impurities coming from the nanorod synthesis (e.g., $Ag^+$), 2 mL solution of gold nanorods used as seeds ([Au(0)] =10 mM) was washed twice with 2 mL of a 10 mM CTAC (purity 99%) aqueous solution, followed by two more washes with chiral co-surfactant/CTAC solution. The nanorods were then redispersed in the chiral co-surfactant/CTAC solution (final [Au(0)] =10 mM) and aged

overnight prior to use. In the case of BINAMINE, a slightly supersaturated solution (maximum CTAC:BINAMINE ratio of 40, excess of 0.1- 0.2 mg/mL) was used for chiral growth, as it provided better reproducibility and higher quality chiral nanoparticles.

- *Preparation of chiral gold nanorods:* In a 2 mL Eppendorf tube, 600 µL of milli-Q water was mixed with 200 µL of ((*R*)- or (*S*)-) BINAMINE/CTAC and 10 µL of a 50 mM Au (III) solution. The mixture was vigorously shaken and allowed to rest at room temperature for five minutes to favor the complexation of Au(III) with CTAC. Then, for the preparation of samples R1, R2, S1, S2, and P2, 13.5 µL of gold nanorod (29 nm in width and 130 nm in length) dispersion and 200 µL of ascorbic acid (0.8 M) were added to the above mixture, which was then shaken vigorously for 5-10 seconds. For R3, the amount of gold nanorod dispersion was increased to 38 µL. The solution was allowed to rest for 10–15 minutes. Finally, it was centrifuged and redispersed in 1 mL of cysteine/CTAC (2mM/10 mM) stock solution (except for P2). P1 was prepared according to the described procedure but using (*R*)- BINOL instead of BINAMINE.

## 4. Tomography procedure

For the reconstruction of the single chiral nanoparticles shown in this manuscript, HAADF-STEM frames were acquired using an aberration-corrected 'cubed' FEI-Titan electron microscope operated at 300 kV with a camera length of 58 mm. The incident electron beam at the implemented acceleration voltage (300 kV) is far from the threshold energy for bulk displacement in Au (1330 kV) and close to the threshold for electron-beam sputtering (270 kV). No beam damage is therefore expected nor observed.[3] Tomography tilt series for each sample were acquired by using a dedicated tomography holder (Fischione 2020) over ±72° with a tilt increment of 3°. The information from each angle consisted of 10 frames of 1k×1k with a scan time of 0.5 s for each frame consecutively. To avoid the possible image distortions, which might hamper the quality of the reconstruction we applied non-rigid registration methods,[4] in combination with a convolutional neural network (CNN)[5,6] in each frame of the time series, effectively removing effect of frequency distortions.

Once the restoration of each frame was accomplished, the restored images were aligned with respect to each other and merged by using phase correlation, which was also used to determine the optimal shift and the angle of the rotation axis. 3D reconstruction was performed by an approach consisting of iteration of SIRT cycles and application of constraints in the real and Fourier spaces.[5] After applying a bandwidth limit to the FFT, the result was transformed into real space and a threshold was applied to the intensity of the 3D volume. Next, the SIRT cycles were repeated. In this manner, a high-quality 3D reconstruction with minimized reconstruction artifacts and errors was obtained.[2]

For the reconstruction shown in Figure 5 of the main text, the tomography series was acquired using a FEI Tecnai Spirit TEM operated at 120 kV under Cryo-TEM conditions. A series of Bright-field TEM images were acquired in a range of ±60° with a tilt increment of 2° using a Fischione 2020 tomography holder. After alignment of the rotation axis the 3D reconstruction of the series was retrieved using the weighted back projection algorithm.[7]

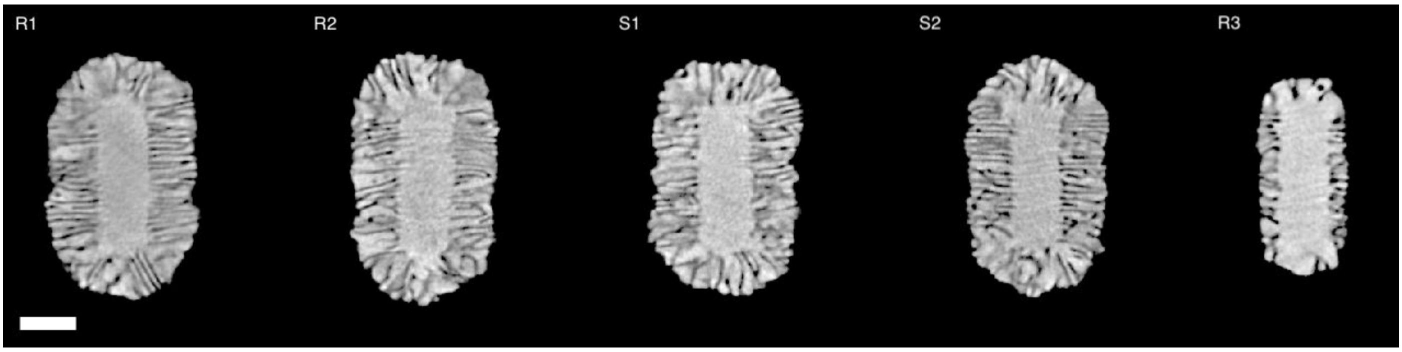


**Figure S3.** Orthoslices of the particle investigated in the main text. The scale bar is 50 nm.

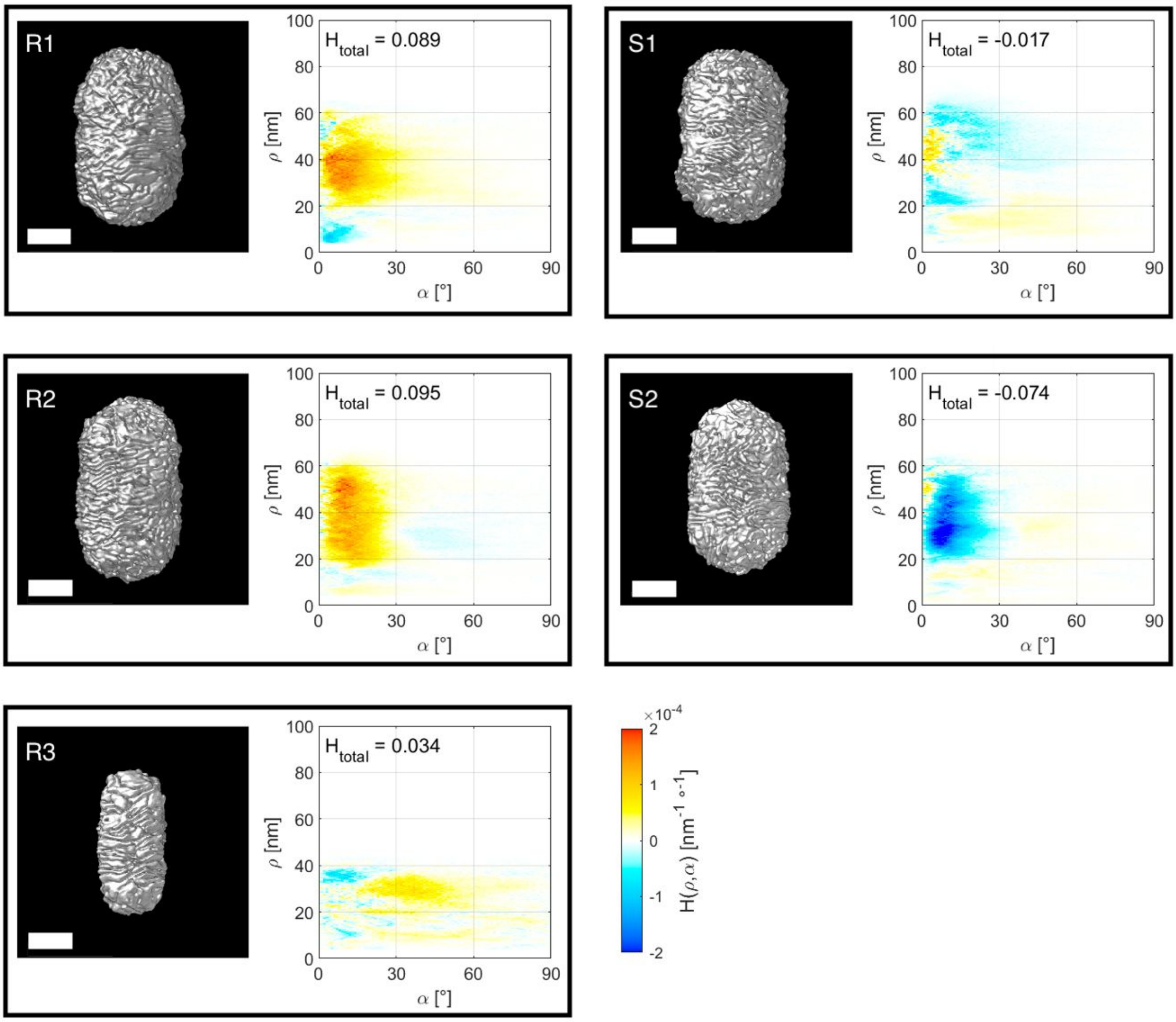


**Figure S4.** Helicity functions for the NRs discussed in the main text, without removing the top and bottom tips of each NR. The helicity functions $H(\rho,\alpha)$ show that the tips add some artifacts to the results and the total helicity $H_{total}$ for each particle is lower when taking the tips into account. Despite quantitative differences compared to Figure 4 in the

main text, the results with and without the tips are qualitatively similar and result in the same handedness. The scale bars are 50 nm.

## 5. Analysis of additional nanorods with poorly defined chirality

To further analyze the results presented in ref. 1, we applied our methodology to two extra particles, described in Table S1. Both particles where synthesized using smaller seeds in comparison to the particles discussed in the main text. P1 was prepared using (*R*)-BINOL/CTAC. P2 was prepared using (*R*)-BINAMINE/CTAC but without adding cysteine after the synthesis, which serves to enhance stability. ET was then performed on P2 after two weeks of storage to observe the deformation that is caused when cysteine is not present. The reconstructions are visualized in Figure S5, as well as the helicity function $H(\rho,\alpha)$ and total helicity $H_{total}$.

We observe that P1 is right-handed helical, which is confirmed by the helicity function $H(\rho,\alpha)$ and the $H_{total}$ value. The helicity function shows that here the inclination angles are more evenly distributed in the range between $\alpha = 0°$ and $\alpha = 90°$. This is an important qualitative difference between the particles created using (*R*)-BINAMINE/CTAC and those created using (*R*)-BINOL/CTAC, which could be a deciding factor when optimizing the synthesis for a certain purpose. It should be noted that $H_{total}$ for P1 is higher than that for R3, although a lower anisotropy factor was reported[1] for the sample containing P1. This could be due to one of two reasons: either P1 is an exceptionally helical particle in its sample, or the different anisotropy factor could be an effect of the difference in the helicity function $H(\rho,\alpha)$. The total helicity $H_{total}$ for P2 is extremely low compared to all other particles investigated. This indicates that it is not helical and confirms that cysteine (or a different stabilizer) was required to maintain the helical shape that was achieved during synthesis. On the other hand, the helicity function $H(\rho,\alpha)$ does show some seemingly significant helicity at low inclination angles. This is due to some holes that are still present inside the NR. Since the NR has few features and is nearly axially symmetric, it also has a small gradient along the cylindrical planes. This leads to a low normalization factor, which increases the intensity in the helicity function $H(\rho,\alpha)$. One should therefore be careful when analyzing particles and always compare the helicity function $H(\rho,\alpha)$ with a visual inspection of the particle and the total helicity $H_{total}$.

**Table S1.** Experimental details of two additional NRs to which our method was applied. P1 was synthesized using (*R*)-BINOL/CTAC instead of (*R*)-BINAMINE/CTAC, and P2 is a NR obtained using (*R*)-BINAMINE/CTAC but on which ET was performed after two weeks of storage without cysteine as a stabilizing agent.

| **Particle** | **Micelle** | **Seed diameter [nm]** | **Seed height [nm]** | **Diameter [nm]** | **Height [nm]** | **Expected handedness** |
|---|---|---|---|---|---|---|
| P1 | (*R*)-BINOL | 32 | 140 | 70 | 192 | Right-handed |
| P2 | (*R*)-BINAMINE | 38 | 140 | 84 | 184 | Achiral |

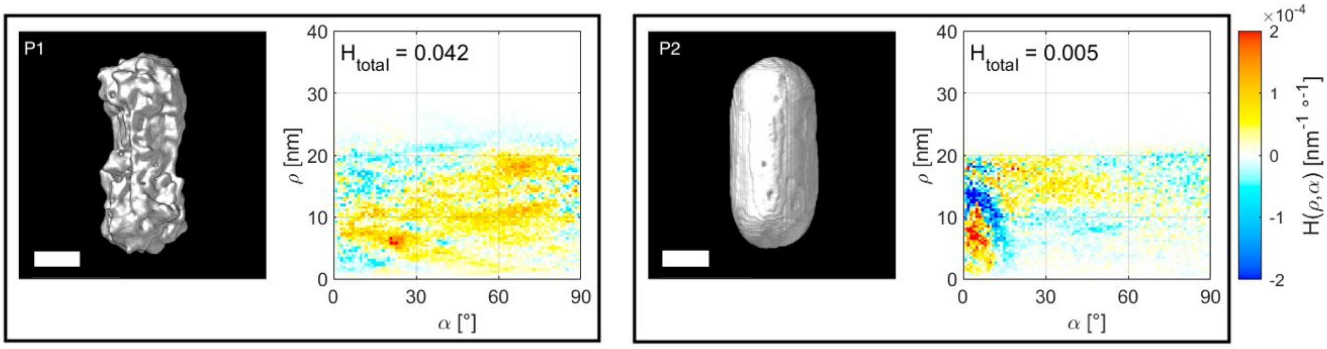


**Figure S5.** Isosurface visualizations of 3D ET reconstructions for Au NRs as described in Table S1 (left image in each panel) along with their helicity function $H(\rho,\alpha)$ and total helicity $H_{total}$ (right image in each panel). The scale bars are 25 nm.

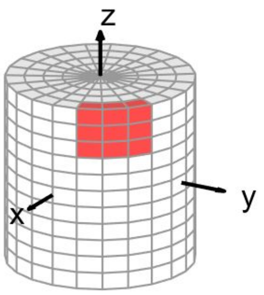


**Figure S6.** Illustration of a window of 3 × 3 voxels (red) on the outer cylindrical section of a 3D cylindrical voxel grid.

### 6. Window size for helicity maps

As mentioned in the main text, there is no objective way to calculate the optimal window size for creating helicity mappings. However, different window sizes can yield vastly different results as shown in Figure S7. If the window size is too large, one can only see the global trend of the NR, while the purpose of creating a helicity map is precisely to analyze local trends. Choosing a window size that is too small will be affected by artifacts and noise in the reconstructions and fail to capture the local helical trend. Therefore, it is up to the researcher to select an optimal window size. An appropriate criterion is to minimize the window size such that only one handedness is assigned to a given helical feature in the particles. For example, in the case of the particles in Figure 3, this means that only one handedness should be assigned to each wrinkle. For our particles, we selected a window size of 32 × 32 voxels based on the results in Figure S7. This choice was made because the features of interest for these particles are the wrinkles observed at the surface. A window size of 32 × 32 voxels corresponds to the smallest window that can distinguish different groups of such wrinkles.

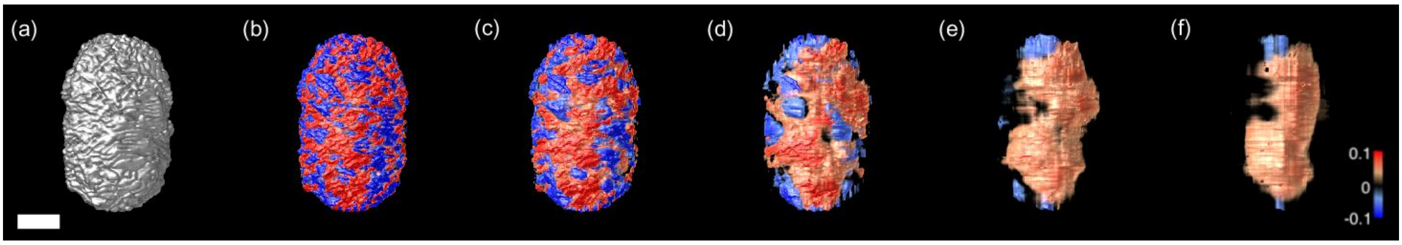


**Figure S7.** 3D volume renderings of the helicity maps for a right-handed helical NR (R1), which is shown as an isosurface in (a), using different window sizes: 8 × 8 voxels (b), 16 × 16 voxels (c), 32 × 32 voxels (d), 64 × 64 voxels (e) and 92 × 92 voxels (f).

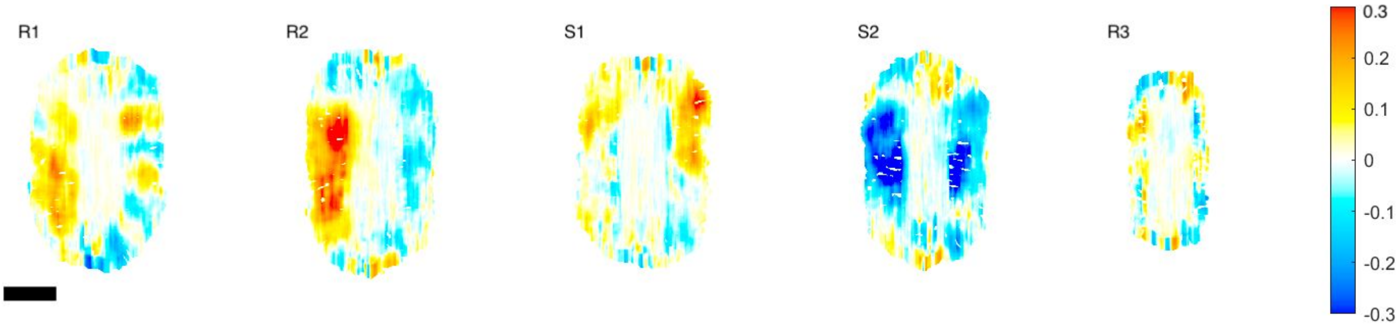


**Figure S8.** Orthoslices through the helicity maps of the particles analyzed in the main text. The scale bar is 50 nm.

## 7. Movies

**Movie S1.** 3D isosurface of the ET reconstruction of particle R1 (left) and a semi-transparent 3D color-coded volume rendering of its helicity map, where red indicates right-handed helicity and blue indicates left-handed helicity. A vertical cut is visible in the particle, this is an artefact caused by converting the volume from a cylindrical to a Cartesian coordinate system but does not affect the results. The well-reconstructed side is visible halfway through the movie.

**Movie S2.** Orthoslices of the ET reconstruction of particle R1 (left) and its helicity map (right). The orthoslices are parallel to the x/y plane. The same cut that is visible in the 3D renderings is also visible on the right-hand side of the orthoslices. The center voxels were left out in the cylindrical voxel grid to reduce artefacts and are therefore also not visible in the orthoslices. These also do not influence the results. The left-hand side of the particle is the well-reconstructed side.

**Movie S3.** 3D isosurface of the ET reconstruction of particle R2 (left) and a semi-transparent 3D color-coded volume rendering of its helicity map, where red indicates right-handed helicity and blue indicates left-handed helicity. A vertical cut is visible in the particle, this is an artefact caused by converting the volume from a cylindrical to a Cartesian coordinate system but does not affect the results. The well-reconstructed side is visible halfway through the movie.

**Movie S4.** Orthoslices of the ET reconstruction of particle R2 (left) and its helicity map (right). The orthoslices are parallel to the x/y plane. The same cut that is visible in the 3D renderings is also visible on the right-hand side of the

orthoslices. The center voxels were left out in the cylindrical voxel grid to reduce artefacts and are therefore also not visible in the orthoslices. These also do not influence the results. The left-hand side of the particle is the well-reconstructed side.

**Movie S5.** 3D isosurface of the ET reconstruction of particle S1 (left) and a semi-transparent 3D color-coded volume rendering of its helicity map, where red indicates right-handed helicity and blue indicates left-handed helicity. A vertical cut is visible in the particle, this is an artefact caused by converting the volume from a cylindrical to a Cartesian coordinate system but does not affect the results. The well-reconstructed side is visible halfway through the movie.

**Movie S6.** Orthoslices of the ET reconstruction of particle S1 (left) and its helicity map (right). The orthoslices are parallel to the x/y plane. The same cut that is visible in the 3D renderings is also visible on the right-hand side of the orthoslices. The center voxels were left out in the cylindrical voxel grid to reduce artefacts and are therefore also not visible in the orthoslices. These also do not influence the results. The left-hand side of the particle is the well-reconstructed side.

**Movie S7.** 3D isosurface of the ET reconstruction of particle S2 (left) and a semi-transparent 3D color-coded volume rendering of its helicity map, where red indicates right-handed helicity and blue indicates left-handed helicity. A vertical cut is visible in the particle, this is an artefact caused by converting the volume from a cylindrical to a Cartesian coordinate system but does not affect the results. The well-reconstructed side is visible halfway through the movie.

**Movie S8.** Orthoslices of the ET reconstruction of particle S2 (left) and its helicity map (right). The orthoslices are parallel to the x/y plane. The same cut that is visible in the 3D renderings is also visible on the right-hand side of the orthoslices. The center voxels were left out in the cylindrical voxel grid to reduce artefacts and are therefore also not visible in the orthoslices. These also do not influence the results. The left-hand side of the particle is the well-reconstructed side.

**Movie S9.** 3D isosurface of the ET reconstruction of particle R3 (left) and a semi-transparent 3D color-coded volume rendering of its helicity map, where red indicates right-handed helicity and blue indicates left-handed helicity. A vertical cut is visible in the particle, this is an artefact caused by converting the volume from a cylindrical to a Cartesian coordinate system but does not affect the results.

**Movie S10.** Orthoslices of the ET reconstruction of particle R3 (left) and its helicity map (right). The orthoslices are parallel to the x/y plane. The same cut that is visible in the 3D renderings is also visible on the right-hand side of the orthoslices. The center voxels were left out in the cylindrical voxel grid to reduce artefacts and are therefore also not visible in the orthoslices. These also do not influence the results.

**Movie S11.** 3D isosurface of the ET reconstruction of particle P1 (left) and a semi-transparent 3D color-coded volume rendering of its helicity map, where red indicates right-handed helicity and blue indicates left-handed helicity.

**Movie S12.** Orthoslices of the ET reconstruction of particle P1 (left) and its helicity map (right). The orthoslices are parallel to the x/y plane. The center voxels were left out in the cylindrical voxel grid to reduce artefacts and are therefore also not visible in the orthoslices. These do not influence the results.

**Movie S13.** 3D isosurface of the ET reconstruction of particle P2 (left) and a semi-transparent 3D color-coded volume rendering of its helicity map, where red indicates right-handed helicity and blue indicates left-handed helicity.

**Movie S14.** Orthoslices of the ET reconstruction of particle P2 (left) and its helicity map (right). The orthoslices are parallel to the x/y plane. The center voxels were left out in the cylindrical voxel grid to reduce artefacts and are therefore also not visible in the orthoslices. These do not influence the results.

**Movie S15.** 3D isosurface of the ET reconstruction of the helical structure of Au NRs clustered around an amyloid fibril (left) and a semi-transparent 3D color-coded volume rendering of its helicity map, where red indicates right-handed helicity and blue indicates left-handed helicity.

**Movie S16.** Orthoslices of the ET reconstruction of the helical structure of Au NRs clustered around an amyloid fibril (left) and its helicity map (right). The orthoslices are parallel to the x/y plane.

**Movie S17.** Aligned tilt series of particle R2.

## 7. References


[1] González-Rubio, G.; Kumar, V.; Llombart, P.; Díaz-Núñez, P.; Bladt, E.; Altantzis, T.; Bals, S.; Peña-Rodríguez, O.; Noya, E. G.; MacDowell, L. G.; Guerrero-Martínez, A.; Liz-Marzán, L. M. Disconnecting Symmetry Breaking from Seeded Growth for the Reproducible Synthesis of High Quality Gold Nanorods. *ACS Nano* **2019**, *13*, 4424–4435.

[2] González-Rubio, G.; Mosquera, J.; Kumar, V.; Pedrazo-Tardajos, A.; Llombart, P.; Solís, D. M.; Lobato, I.; Noya, E. G.; Guerrero-Martínez, A.; Taboada, J. M.; Obelleiro, F.; MacDowell, L. G.; Bals, S.; Liz-Marzán, L. M. Micelle-Directed Chiral Seeded Growth on Anisotropic Gold Nanocrystals. *Science* **2020**, *368*, 1472–1477.

[3] Egerton, R. F. Choice of operating voltage for a transmission electron microscope. *Ultramicroscopy* **2014,** *145*, 85-93.

[4] Jones, L.; Yang, H.; Pennycook, T. J.; Marshall, M. S. J.; Van Aert, S.; Browning, N. D.; Castell M. R.; Nellist, P. D. Smart Align - a new tool for robust non-rigid registration of scanning microscope data. *Adv. Struct. Chem. Imaging* **2015,** *1*, 8.

[5] Altantzis, T.; Lobato, I.; De Backer, A.; Béché, A.; Zhang, Y.; Basak, S.; Porcu, M.; Xu, Q.; Sánchez-Iglesias, A.; Liz-Marzán, L. M.; Van Tendeloo, G.; Van Aert, S.; Bals, S. Three-Dimensional Quantification of the Facet Evolution of Pt Nanoparticles in a Variable Gaseous Environment. *Nano Lett.* **2019,** 19, 477–481.

[6] Krizhevsky, A.; Sutskever, I.; Hinton, G. E. Imagenet classification with deep convolutional neural networks. *Adv. Neural Inf. Process. Syst.* **2012,** *25*, 1097–1105.

[7] Kumar, J.; Eraña, H.; López-Martínez, E.; Claes, N.; Martín, V. F.; Solís, D. M.; Bals, S.; Cortajarena, A. L.; Castilla, J.; Liz-Marzán, L. M. Detection of amyloid fibrils in Parkinson's disease using plasmonic chirality. *Proc. Natl. Acad. Sci.* **2018,** *115*, 3225–3230.
